# High *MET* gene copy number predicted poor prognosis in primary intestinal diffuse large B-cell lymphoma

**DOI:** 10.1186/1746-1596-8-16

**Published:** 2013-02-04

**Authors:** Wan-Ting Huang, Shih-Sung Chuang

**Affiliations:** 1 Department of Pathology, Kaohsiung Chang Gung Memorial Hospital and Chang Gung University College of Medicine, Kaohsiung, Taiwan; 2 Department of Pathology, Chi-Mei Medical Center, Tainan, Taipei Medical University and National Taiwan University Hospital, Taipei, Taiwan

**Keywords:** Copy number, Diffuse large B-cell lymphoma, Intestine, *MET*, Primary intestinal diffuse large B-cell lymphoma, Taiwan

## Abstract

**Background:**

*MET* is a proto-oncogene with its copy number (CN) alterations been reported in some cancers, but not in primary intestinal diffuse large B-cell lymphoma (PI-DLBL) yet.

**Methods:**

In this retrospective study, we performed histology and chart reviews, immunohistochemistry and quantitative polymerase chain reaction for *MET* CN alterations on 28 surgically resected PI-DLBLs.

**Results:**

There were 12 men and 16 women with a median age of 70 and a mean follow-up of 32 months. The median *MET* CN was 2.20 (range, 1.04 to 3.35). CN gain was observed in 11 cases, including 5 with CN greater than 3. Nine patients (32%) had diploid CN and eight (29%) with CN loss. Patients with gain or diploid CN showed significantly worse prognosis (*P* = 0.046) than those with CN loss. Furthermore, *MET* CN greater than 3 was associated with an adverse outcome (*P* = 0.003). Intestinal perforation at presentation was the sole clinicopathological factor associated with a poor prognosis (*P* = 0.004) and perforation was correlated with CN greater than 3 (*P* = 0.002).

**Conclusions:**

Our finding of *MET* CN gain as a poor prognostic factor in PI-DLBL patients might serve as the rationale for targeting *MET* signaling pathway in the treatment of these patients.

## Background

Copy number alterations (CNAs) of genes are indications of genomic instability that are common in cancers and have been known to be associated with the development of malignancy [1,2]. CNAs of specific genes may deregulate signal pathways that enhance the proliferation and inhibit the apoptosis of tumor cells. Cumulative evidences suggest a link between CNAs and prognosis of cancer patients [3]. CNAs of particular genes may provide insights into the pathogenesis of certain neoplasms and the clues for target therapy. Hepatocyte growth factor (HGF)/MET pathway is one of the most commonly activated signaling pathways in human malignancies. Deregulated HGF/MET signaling axis contributes to tumorigenesis and invasion/metastasis by the following cellular behaviors collectively described as invasive growth: proliferation and survival (resistance to apoptotic signals), increased cell motility, cell dissociation (scattering), epithelial tubulogenesis, infiltration of tissues, and stimulation of angiogenesis [4-6]. The HGF/MET pathway may be activated by mutations or genomic amplification of the *MET* gene and ligand-independent kinase activation by extracellular proteins, autocrine overexpression of HGF ligand, and increased expression of HGF activator [6,7]. Recent studies have provided important evidence for *MET* CNAs in gastric cancer and non-small cell lung cancer (NSCLC) [8-10]. Although some studies have shown that HGF/MET pathway might be implicated in B-cell neoplasms particularly diffuse large B-cell lymphoma (DLBL) and multiple myeloma (MM) [11-18], it is still an unresolved issue for the impact of *MET* CNA in the survival of patients with these hematological malignancies.

In a previous study, we identified perforation as the sole clinicopathological prognostic factor in patients with primary intestinal DLBL (PI-DLBL) [19]. In this current retrospective study we investigated the prognostic impact of *MET* CNAs by quantitative polymerase chain reaction (qPCR) method and found that in addition to perforation, *MET* CNAs were also significantly associated with prognosis.

## Materials and methods

### Case selections and clinical data

Strict inclusion and diagnostic criteria for PI-DLBL were applied as previously reported [19]. Medical records were reviewed and overall survival was measured from the date of diagnosis to the date of last follow-up. Chi-Mei Institutional Review Board approved this study.

### DNA extraction

To avoid the contamination by normal cells, we reviewed the HE sections of each case and chose those containing greater than 90% tumor cells without or with only minimal tumor necrosis for DNA extraction. Three to five 10 μm thick paraffin sections of each case were cut into an eppendorf tube from a representative block with disposable blades. The paraffin rolls were de-paraffinized with xylene followed by three washings with ethanol. Paraffin-free tissue was dried in a heating box for 15 min at 37°C and then subjected to the DNA extraction procedures using QIAamp DNA mini kit (Qiagen, Hilden, Germany). The quality and DNA concentration were measured by NanoPhotometer (IMPLEN, München, Germany). DNA with 260/280 ratios of 1.6–2.0 with a minimal concentration of 25 ng/μl DNA were required for qPCR assay.

### Copy number assay

We performed qPCR assay to determine the CNAs of *MET* gene by using the commercially available 6-carboxy-fluorescine (FAM)-labeled *MET* probe (Applied Biosystems assay ID no. Hs02764674, Applied Biosystems, Foster City, CA). Ribonuclease P (RNase P), which is known to exist in two copies, was used as the endogenous control with 4,7,2-trichloro-7-phenyl-6-carboxyfluorescein (VIC)-labeled probe (Applied Biosystems Part no. 4403326). The peripheral blood mononuclear cells from six healthy individuals served as control samples. Each experiment was performed using a 96-well optical PCR plate and the StepOnePlus real-time PCR machine (Applied Biosystems) with default cycling conditions. According to manufacturer’s instruction, a total of 10 ng of genomic DNA was added to the PCR reaction mixture containing 1× final concentration TaqMan Gene Expression PCR Master Mix (Applied Biosystems) and 1x final pre-designed primer/probe Mix (Applied Biosystems) in a final volume of 10μL. Each sample was run in triplicate. After amplification, the Ct values for both target and reference genes were imported into the CopyCaller Software (Applied Biosystems) for data analysis. The CN of the target gene was determined by comparative quantitative threshold cycle (ΔΔCt) method where ΔΔCt = (Ct of target gene, test sample − Ct of RNase P, test sample) − (average Ct of target gene, reference samples − average Ct of RNase P, reference samples) and then using the formula 2 × 2^− ΔΔCt^[20]. The cutoff value of target genes was set at 3 standard deviation of the mean derived from the six normal control samples. Each sample was then assigned to have diploid *MET* CN, gain, or loss.

### Statistical analysis

The associations between *MET* CN and various clinical and immunophenotypical parameters were assessed by *χ*2 test or Fisher’s exact test. Kaplan–Meier survival curves were drawn and the log rank test was used to compare the differences between survival curves. All statistical analyses were performed using SPSS for windows 11.0 software (SPSS Inc. Chicago, IL). A *P* value of 0.05 or less was considered statistically significant.

## Results

**Figure 1:**
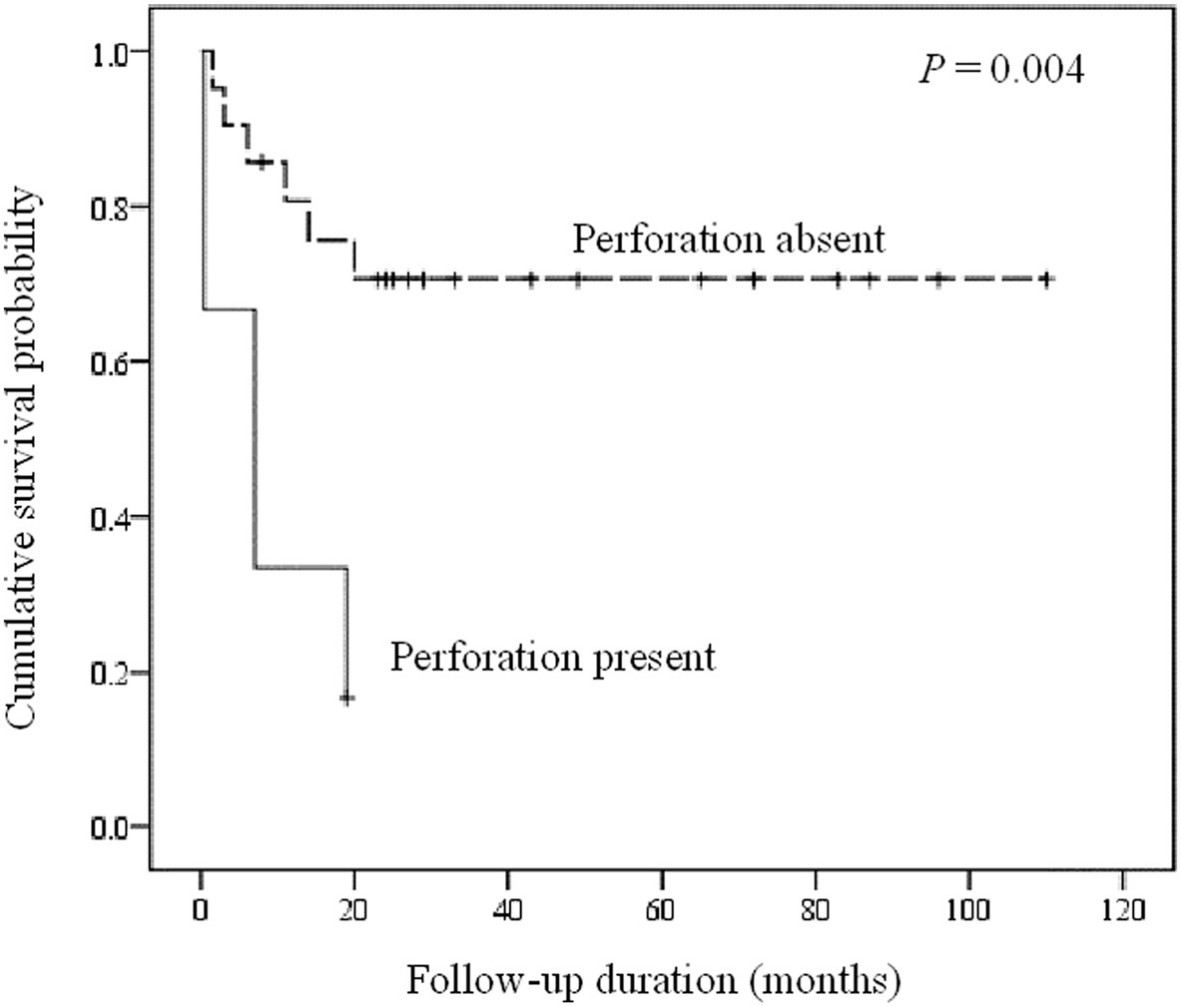
Cumulative survival shows a significantly poorer outcome in patients presented with perforation.

These 28 patients included 12 males and 16 females with a median age of 70 years (range, 26 to 88). Six patients (21%) presented with intestinal perforation. The staging results were available in 27 patients including 9 (33%) at stage IE, 14 (52%) at stage IIE and 4 (15%) at stage IVE. Nineteen patients (70%) received chemotherapy with cyclophosphamide, doxorubicin, vincristine, and prednisolone (CHOP) or similar regimens. Among these, 12 patients received combined Rituximab therapy and others additional radiotherapy. Eight patients (30%) received only supportive treatment without chemo- or radiotherapy due to poor general condition. The mean follow-up time was 32 months (range, 0.3 to 110). The overall 1-, 2- and 5-year survival rates were 50, 43 and 18%, respectively. Immunohistochemically, 22 tumors (79%) expressed bcl-2, bcl-6 and MUM1/IRF4; while only 6 tumors (21%) expressed CD10. Nineteen tumors (68%) had concurrent expression of bcl-2 and MUM1. All tumors were negative for cyclin D1. Seven tumors (25%) were classified as germinal centre B-cell (GCB) phenotype and 21 non-GCB (75%) according to the algorithm of Hans et al. [21]. Patients with intestinal perforation at presentation showed a poor prognosis compared to those without (*P* = 0.004; Figure 1). The other clinicopathological or immunohistochemical findings including all differentiation markers and GCB vs. non-GCB phenotype were of no prognostic significance.

**Figure 2:**
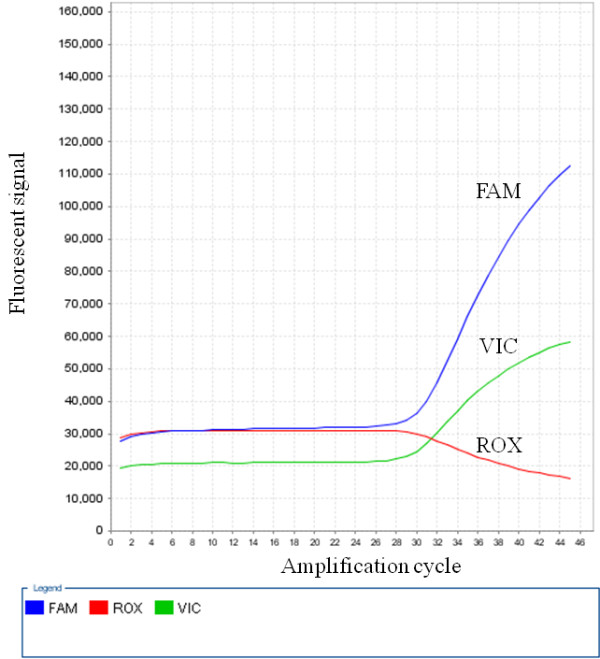
**A representative case with MET copy number data analyzed by CopyCaller Software showing increased FAM to VIC ratio, indicating increased CN.** Abbreviations: FAM, 6-carboxy-fluorescine-labeled *MET* probe; ROX (carboxy-X-rhodamine), internal reference dye used for normalization of fluorescent signal variations between wells; VIC: 4,7,2-trichloro-7-phenyl-6-carboxyfluorescein-labeled *RNase P* probe.

**Figure 3:**
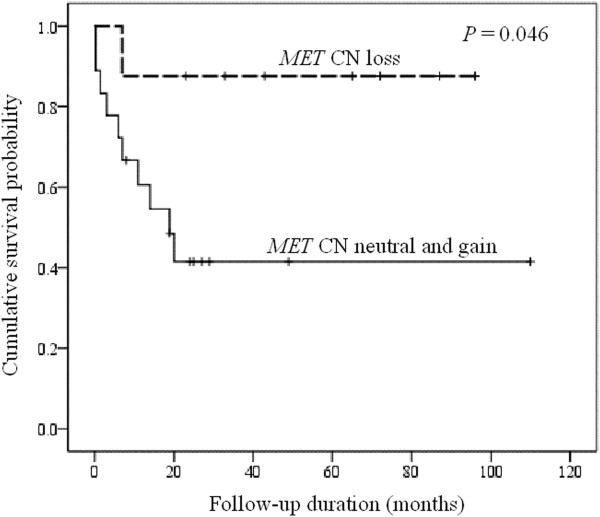
**Cumulative survival shows a significantly poorer outcome in patients with diploid or gain of *MET *CN than those with CN loss.**

**Figure 4:**
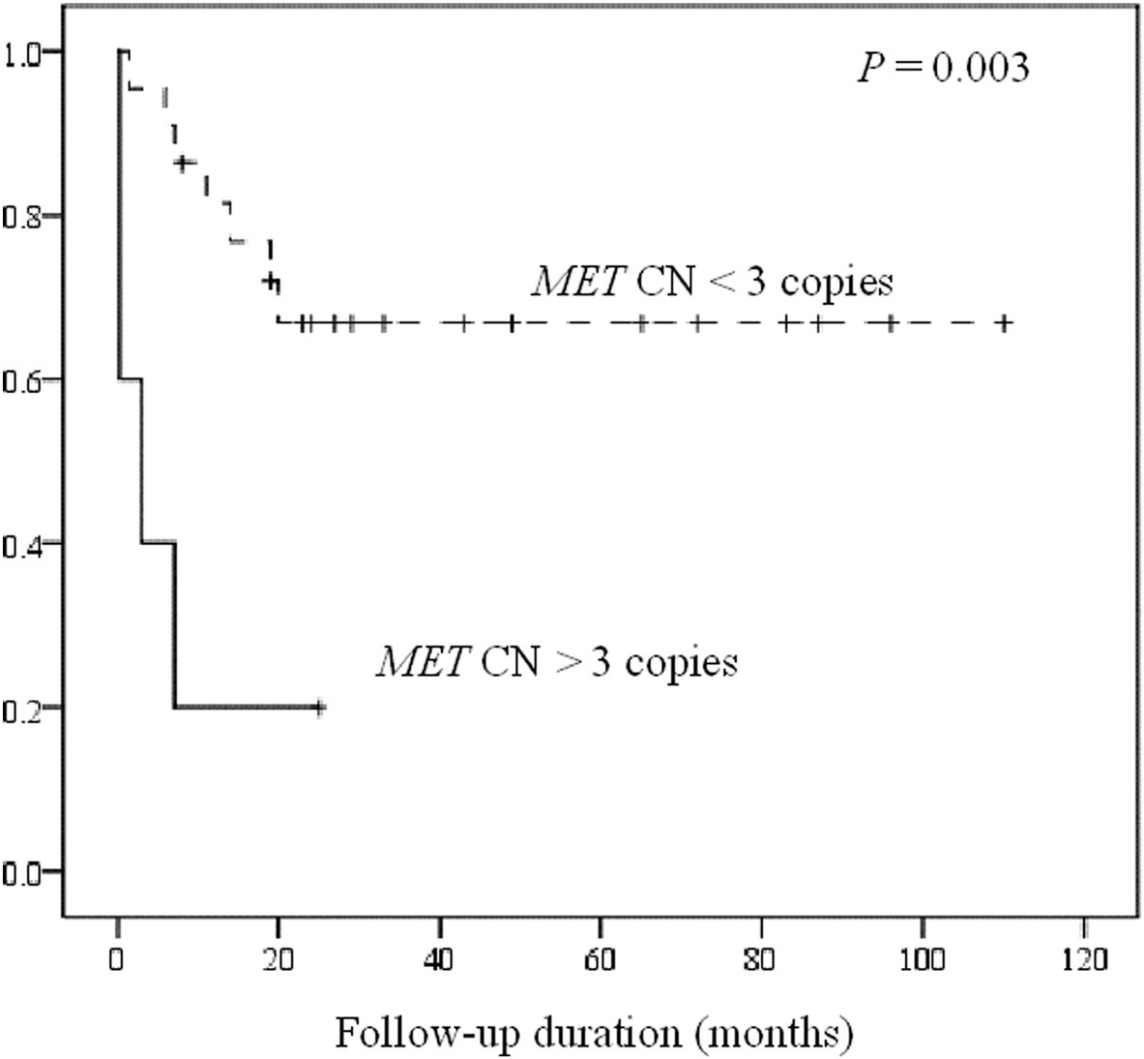
**Cumulative survival shows a significantly poorer outcome in patients with *MET *CN > 3 copies than those with CN < 3 copies.**

Table 1 summarizes the associations between *MET* CN and the clinicopathologic features. Of these 28 cases the median *MET* CN was 2.20 (range, 1.04 to 3.35; Figure 2). Deriving from the results of normal control specimens, the tumors with a cut-off value of greater than 2.35 copies was designated as having *MET* CN gain, and those with less than 1.77 as CN loss. According to these criteria, 11 patients (39%) had *MET* CN gain (median: 2.94 copies; range, 2.48 to 3.35), 8 (29%) with CN loss (median: 1.33; range, 1.04 to 1.66), and 9 (32%) with normal/diploid CN (median: 2.00; range, 1.91 to 2.28). Patients with *MET* CN gain or normal/diploid *MET* CN showed a significant worse prognosis than those with *MET* CN loss (*P* = 0.046; Figure 3). We also found that the patients with *MET* CN gain of greater than 3 copies showed a poorer prognosis as compared to those with CN of less than 3 (*P* = 0.003; Figure 4). There was also a significant correlation between *MET* CN gain of greater than 3 copies and perforation at disease presentation (*P* = 0.002).

**Table 1:** **Correlation between *MET *copy number and parameters**

		***MET *copy number**			
	**Case no. (%)**	**>3 copies (%)**	***P***	**Loss (%)**	***P***
Gender					
Male	12 (43)	2 (40)	0.89	3 (38)	0.72
Female	16 (57)	3 (60)		5 (62)	
Age (years)					
≥60	8 (29)	1 (20)	0.65	2 (25)	0.8
<60	20 (71)	4 (80)		6 (75)	
B symptom					
Present	6 (22)	1 (20)	0.9	0 (0)	0.08
Absent	21 (78)	4 (80)		8 (100)	
Perforation					
Present	6 (21)	3 (60)	0.02	1 (13)	0.48
Absent	22 (79)	2 (40)		7 (87)	
Ann Arbor stage					
I or II	23 (85)	5 (100)	0.31	7 (87)	0.83
III or IV	4 (15)	0 (0)		1 (13)	
Immunophenotype					
GCB	7 (25)	1 (20)	0.78	3 (38)	0.34
Non-GCB	21 (75)	4 (80)		5 (62)	

## Discussion

In this study, we investigated the prognostic role of the *MET* CNAs in surgically resected PI-DLBL and found that tumors with *MET* CN gain greater than 3 copies were associated with an unfavorable prognosis and also with perforation at disease presentation. Normal or lower level *MET* CN gain also denoted a trend toward poor prognosis, although the statistical significance was low. We speculate that the clinical impact of *MET* CN on prognosis might be amplification dosage dependent. Furthermore, we re-confirmed our previous findings that tumor perforation was a poor prognostic factor in PI-DLBL and that the relative frequency of GCB phenotype was low in PI-DLBL, although the latter was not significantly related to prognosis [19].

HGF/MET pathway could be activated in both benign and malignant B-cells. Amplification of the *MET* gene would result in MET protein over-expression and constitutive kinase activation [22]. *MET* CNAs have been investigated in solid cancer such as gastric carcinoma and NSCLC [8-10]. Depending on the methods for assessing *MET* CNAs, *MET* amplification has been identified in 4-21% of gastric carcinomas [23,24]. An increase of *MET* CN was associated with higher tumor stage at presentation and poorer survival in patients with gastric cancer [10,25]. For a subset of lung cancers with acquired resistance to epidermal growth factor receptor-targeting therapy, *MET* amplification is one of the most frequently involved mechanisms [26]. In NSCLC, *MET* gene has been shown to be amplified in 21-24% of tumors and the impact of *MET* CN on prognosis is histological subtype dependent: *MET* amplification with a poorer prognosis in patients with adenocarcinoma but not in those with squamous cell carcinoma [9,27].

Among the various entities of non-Hodgkin lymphomas, MET is frequently over-expressed in DLBL [14,15,17]. Over-expression of HGF/MET signaling activates downstream molecules that control proliferation, adhesion and apoptosis. In the literature, however, the prognostic significance of MET over-expression in DLBL is controversial. High serum HGF levels and overactive HGF/MET pathway in DLBL patients have been reported to be linked with unfavorable outcome in several studies [14,28,29]. On the other hand, Uddin et al. showed a better overall survival at 5 years in DLBL patients with MET over-expression than those with low MET expression level (76.2% vs. 57.5%; *P* = 0.0025) [13]. A study from the Middle East also showed that MET over-expression carried a better prognosis in DLBL patients [13]. So far *MET* gene CN has not been investigated in malignant lymphoma yet. To the best of our knowledge, our study is the first report describing the prognostic significance of *MET* CNAs in patients with PI-DLBL. Our results suggested that higher *MET* CN, possibly leading to MET over-expression, might be a poor prognostic indicator for PI-DLBL.

High serum HGF levels have been found in patients with DLBL and MM, and they were associated with an unfavorable prognosis [14,30-32]. Furthermore, tumor cells could up-regulate HGF production, through paracrine and/or autocrine mechanisms, by the surrounding stromal cells or cancer cells themselves [7,33]. With the recent development of clinical-grade agents targeting the HGF/MET pathway, inhibition of this pathway is currently considered a rational and promising strategy for the treatment of patients with B-cell lymphoma and MM [13,34]. Among these agents, Crizotinib (PF-02341066) is an orally bioavailable, ATP-competitive, small-molecule inhibitor of c-MET and anaplastic lymphoma kinase, and is considered a very potential agent for treatment of cancers dependent on these oncogenic kinases for growth and survival [35,36]. Our results of high *MET* CN gain in PI-DLBL patients with poor prognosis suggested that blocking HGF/MET pathway might be a promising adjuvant therapeutic option.

## Abbreviations

CN: Copy number;PI-DLBL: Primary intestinal diffuse large B-cell lymphoma;CNA: Copy number alteration;HGF: Hepatocyte growth factor;NSCLC: Non-small cell lung cancer;DLBL: Diffuse large B-cell lymphoma;MM: Multiple myeloma

## Competing interests

The authors declare that they have no competing interests.

## Authors’ contributions

WTH did the experiment, analyzed the data and drafted the manuscript. SSC conceived and coordinated the study, analyzed the data and wrote the manuscript. Both authors read and approved the final manuscript.
